# Cholesterol, high-density lipoprotein, glucose index as a novel marker for predicting in-stent restenosis after drug-eluting stent implantation in patients with acute coronary syndrome

**DOI:** 10.3389/fcvm.2026.1740216

**Published:** 2026-02-03

**Authors:** Yixiong Lin, Jiaxing Ke, Shuling Chen, Jinghan Yang, Chenxin Liao, Feng Peng, Dajun Chai, Jinxiu Lin

**Affiliations:** 1 Department of Cardiology, The First Affiliated Hospital, Fujian Medical University, Fuzhou, China; 2 The Higher Educational Key Laboratory for Cardiovascular Disease of Fujian Province, Clinical Research Center for Metabolic Heart Disease of Fujian Province, The First Affiliated Hospital, Fujian Medical University, Fuzhou, China; 3 Department of Cardiology, National Regional Medical Center, Binhai Campus of the First Affiliated Hospital, Fujian Medical University, Fuzhou, China

**Keywords:** acute coronary syndrome, biomarker, cholesterol, high-density lipoprotein, glucose index, drug-eluting stent, in-stent restenosis

## Abstract

**Background:**

Acute coronary syndrome (ACS) poses a serious health risk, and drug-eluting stent (DES) implantation is widely used to improve prognosis. However, the risk of in-stent restenosis (ISR) persists in some patients. The CHG index, a novel metabolic marker, has not been clearly linked to ISR risk in ACS patients undergoing DES-based percutaneous coronary intervention (PCI).

**Methods:**

This retrospective study enrolled ACS patients who underwent PCI with successful DES implantation from June 2015 to July 2021 and and underwent coronary angiography at 6 to 24 months after successful DES-based PCI. Patients were stratified into tertiles based on CHG index. Logistic regression analysis models were used to evaluate the independent association between CHG index and ISR. Restricted cubic spline (RCS) models were used to examine potential nonlinear relationships, and subgroup analyses explored possible effect modifiers.

**Results:**

A total of 454 patients with ACS were included. In the fully adjusted model, CHG index was positively associated with DES-ISR incidence (per 1-unit increase, odds ratio [OR] = 2.61, 95% confidence interval [CI] 1.28–5.33, *P* = 0.008). Compared to the lowest tertile, the ORs (95% CI) for the second and third tertiles were 2.33 (1.12–4.85, *P* = 0.024) and 2.40 (1.05–5.49, *P* = 0.038), respectively. Furthermore, a linear positive association was observed between CHG index and risk of ISR post-PCI (overall *P* = 0.016; nonlinear *P* = 0.118).

**Conclusion:**

For ACS patients treated with DES-PCI, a high CHG index was found to be significantly and linearly associated with an increased risk of DES-ISR.

## Introduction

Despite advances in drug-eluting stents (DES) and secondary prevention, in-stent restenosis (ISR) remains a significant challenge, occurring in approximately 10% of percutaneous coronary interventions (PCI) (1). Of additional concern, the treatment of patients with DES-ISR has proven to be particularly challenging (2). Therefore, identifying risk factors for ISR following DES implantation is essential for optimizing personalized therapy and enhancing secondary prevention (3, 4).

Composite metabolic indices are gaining attention for capturing multifaceted cardiometabolic risk. The CHG index, derived from total cholesterol (TC), fasting blood glucose (FBG), and high-density lipoprotein cholesterol (HDL-C), integrates lipid and glucose metabolism into a single marker (5). Each component independently correlates with cardiovascular risk (6), elevated TC accelerates atherogenesis (7), elevated FBG drives endothelial dysfunction through inflammation and oxidative stress (8), and reduced HDL-C impairs reverse cholesterol transport and anti-inflammatory activity, weakening vascular protection (9). Thus, the CHG index may offer a more holistic risk assessment than its individual components. Although elevated CHG index correlates with cardiovascular events, its role in predicting ISR after DES implantation in ACS patients is unclear (5, 10).

Given the pro-atherogenic effects of high TC and FBG, coupled with the loss of vasoprotection from low HDL-C, the CHG index likely reflects a metabolic milieu conducive to neointimal hyperplasia and restenosis. Therefore, investigating its association with ISR could reveal novel aspects of residual metabolic risk and aid in early high-risk identification post-ACS.

## Patients and methods

### Study population

This was a retrospective observational study. A total of 454 patients diagnosed with ACS in the First Hospital of Fujian Medical University, were enrolled from June 2015 to September 2021 who underwent coronary angiography at 6 to 24 months after successful DES-PCI. The following exclusion criteria were as follows, (1) age less than 18 years; (2) history of coronary artery bypass grafting (CABG) and stent implantation; (3) no DES implantation; (4) critical structural heart disease requiring intervention; (5) severe liver, respiratory and renal dysfunction (estimated glomerular filtration rate [eGFR] < 30 mL/min/1.73 m^2^); (6) advanced malignant tumors with short life expectancy; (7) incomplete clinical data or death during hospitalization. The study was approved by the local Ethics Committee and adhered to the Declaration of Helsinki. Informed consent was waived due to the retrospective, anonymized nature of the data.

### Intervention and management

The coronary intervention and perioperative management were performed in accordance with the current guidelines of our centre (11). All patients received guideline-directed medical therapy, including antiplatelet, lipid-lowering, and glucose-lowering agents. Radial access was preferred. DES type, stent length, diameter, and number were at the operator's discretion. Dual antiplatelet therapy (aspirin combined with clopidogrel or Tegretol) for a minimum of 12 months was recommended in all cases afer the implantation of a drug-eluting stent (DES-PCI), and long-term medication management was guided by individual risk.

### Data collection and definitions

Patient demographic, medical history, and clinical characteristics, including age, sex, smoking status, left ventricular ejection fraction (LVEF), angiographic evaluation results, interventional parameters such as number of stents, lesion type, and target vessel, as well as discharge medications, were collected from the electronic medical record system. Furthermore, peripheral venous blood was collected at least 8 h of fasting and analyzed for fasting blood glucose(FBG), uric acid, estimated glomerular filtration rate(eGFR), C-reactive protein (CRP), lipid profile, including triglycerides (TG), TC, LDL-C, and HDL-C.

The definition of ACS complied with the current guideline of the European Society of Cardiology (12). Diabetes mellitus was defined as a prior specialist diagnosis, current use of glucose-lowering therapy, or the presence of diabetic symptoms with either random plasma glucose ≥11.1 mmol/L, fasting plasma glucose ≥7.0 mmol/L, or 2 h plasma glucose ≥11.1 mmol/L during a 75-g oral glucose tolerance test (13). Hypertension was defined as a prior diagnosis by a physician, current use of antihypertensive therapy, or systolic blood pressure ≥140 mmHg and/or diastolic blood pressure ≥90 mmHg on at least three separate occasions (14). Hypercholesterolemia was defined as fasting serum TC ≥ 6.22 mmol/L, fasting LDL-C ≥ 4.14 mmol/L, or current lipid-lowering therapy (15). Hyperuricemia was defined as serum uric acid ≥420 μmol/L in males or ≥360 μmol/L in females (16). Smoking status was categorized as current smoking or non-smoking, and alcohol consumption as current drinking or non-drinking. Body mass index (BMI) was calculated as weight in kilograms divided by height in meters squared (kg/m^2^) (17). Based on angiographic assessment, multi-vessel disease was defined as the presence of at least two vessels with significant diameter stenosis (≥50%), while multi-stent implantation was defined as placement of two or more stents (18). The equation used to calculate eGFR (mL/min/1.73 m^2^) is as follows, 186 × SCr (mg/dL)^(−1.154)^ × age^(−0.203)^ × 0.742 (if female) (19). The Gensini score was determined according to both the degree and location of coronary stenosis, stenosis severity of 25%, 50%, 75%, 90%, 99%, and complete occlusion corresponded to scores of 1, 2, 4, 8, 16, and 32, respectively, each multiplied by a segment-specific weighting factor, with all segment scores summed to yield the final score (20). The Atherogenic Index of Plasma (AIP) was calculated as the logarithm of the ratio between triglycerides (TG) and high-density lipoprotein cholesterol (HDL-C). The formula for calculating the metabolic score for insulin resistance(METS-IR) index was: Ln [2 × FBG (mg/dL) +TG (mg/dL)] × BMI/Ln[ HDL-C (mg/dL)] (21). The formula for calculating the TyG index was: Ln [TG (mg/dL) × FBG (mg/dL)/2] (22). The formula for calculating the CHG index was: CHG index = Ln [TC (mg/dL) × FBG (mg/dL)/2 × HDL (mg/dL)] (10).

### Follow-up angiography and assessment of ISR

All patients underwent scheduled outpatient follow-up, with follow-up coronary angiography performed 6 to 24 months after successful DES-PCI. On the basis of angiographic follow-up results, patients were categorized into ISR and non-ISR groups, which was defined as the presence of significant diameter stenosis (≥50%) at the segment inside the stent or involving its 5 mm edges, which is in line with previous studies (23). Of note, the follow-up angiography was determined by experienced interventional cardiologists.

### Statistical analysis

Continuous variables were presented as mean ± standard deviation (x ± s) for normally distributed data or as median and interquartile range for non-normally distributed data. Categorical variables were described as frequency counts and percentages (%). Group differences were assessed using one-way analysis of variance (ANOVA) for normally distributed continuous variables, the Kruskal–Wallis test for non-normally distributed variables, and the chi-square (*χ*^2^) or Fisher's exact test for categorical variables.

The predictive performance of the CHG index for drug-eluting stent in-stent restenosis (DES-ISR) was evaluated using receiver operating characteristic (ROC) analysis. The area under the curve (AUC) and the optimal cutoff value were derived from this analysis. Univariate logistic regression identified potential predictors of ISR after successful DES-PCI. Variables with a univariate *P*-value <0.1 (Supplementary Table S1) or those deemed clinically relevant were entered into multivariate logistic regression models (4, 11, 23). Three models were developed to mitigate confounding and assess the association between the CHG index (analysed continuously or categorically) and DES-ISR: Model 1 (age, sex, BMI); Model 2 (Model 1 + eGFR, LVEF, CRP, smoking, hypertension, diabetes); Model 3 (Model 2 + Gensini score, multi-vessel disease, multi-stent implantation, total stent length, mean stent diameter). Restricted cubic spline analysis explored the dose-response relationship. Subgroup analyses tested consistency across clinical strata.

To address key confounding, a series of sensitivity analyses were performed to evaluate the robustness of the primary association. Based on the primary fully-adjusted model(Model 3), further adjusting for: (1) statin intensity (high vs. non-high intensity), (2) ezetimibe use, (3) P2Y12 inhibitor type (ticagrelor vs. clopidogrel), and (4) statin intensity and type of P2Y12 inhibitor, and (5) all four medication variables combined.

The CHG index's discriminative performance was compared to other indices (TyG, AIP, METS-IR) using DeLong's test. Model calibration was evaluated with the Hosmer-Lemeshow test. Correlations between the CHG index and cardiovascular risk factors were examined using Pearson or Spearman tests.

All statistical analyses were conducted using SPSS version 27.0 (IBM, Armonk, NY, USA) and R programming language version 4.5.0, with two-sided *P* < 0.05 considered statistically significant.

## Results

### Baseline characteristics

A total of 454 patients who underwent follow-up coronary angiography 6 to 24 months after successful DES-PCI were enrolled. As shown in Table 1, the mean age of the cohort was 63.01 ± 10.25 years, and 370 (81.50%) participants were male. The prevalence of current smoking, hypertension, Hypercholesteraemia, and diabetes mellitus were determined to be 58.59%, 71.37%, 22.25%, and 45.37%, respectively. Regarding angiographic findings, multivessel lesions (71.59%) and PCI for the left anterior descending artery disease (67.62%) was the most common. More than half (57.93%) of the patients had multiple stents (≥2) implanted. Overall, 70 patients (15.12%) experienced ISR.

**Table 1:** Baseline characteristics of patients stratified by tertile of CHG index.

Variables	Total (*n* = 454)	T1 (*n* = 151)	T2 (*n* = 151)	T3 (*n* = 152)	*p*
Age, years	63.01 ± 10.25	65.41 ± 10.48	62.14 ± 9.63	61.49 ± 10.24	0.002
Male, *n* (%)	370 (81.50)	122 (80.79	122 (80.79)	126 (82.89)	0.863
BMI, kg/m^2^	24.15 ± 2.83	23.67 ± 2.99	24.65 ± 2.68	24.14 ± 2.73	0.010
Smoking, *n* (%)	266 (58.59	90 (59.60)	91 (60.26)	85 (55.92)	0.710
Drinking, *n* (%)	156 (34.36)	44 (29.14)	57 (37.75)	55 (36.18)	0.244
Hypertension, *n* (%)	324 (71.37)	108 (71.52)	106 (70.20)	110 (72.37)	0.915
Hypercholesteraemia, *n* (%)	101 (22.25)	18 (11.92)	35 (23.18)	48 (31.58)	<0.001
Diabetes mellitus, *n* (%)	206 (45.37)	34 (22.52)	50 (33.11)	122 (80.26)	<0.001
Previous stroke, *n* (%)	28 (6.17)	13 (8.61)	7 (4.64)	8 (5.26)	0.304
hyperuricemia, *n* (%)	23 (5.07)	5 (3.31)	8 (5.30)	10 (6.58)	0.426
LVEF	61.90 (55.44, 67.75)	64.01 (58.31, 68.62)	62.37 (56.41, 67.40)	59.88 (52.36, 66.40)	0.003
Laboratory tests					
LDL, mmol/L	3.02 ± 1.07	2.56 ± 0.76	3.03 ± 0.96	3.48 ± 1.22	<0.001
TC, mmol/L	4.43 ± 1.10	3.99 ± 0.84	4.42 ± 0.94	4.89 ± 1.27	<0.001
HDL, mmol/L	0.99 ± 0.26	1.13 ± 0.29	0.98 ± 0.22	0.87 ± 0.20	<0.001
Triglyceride, mmol/L	1.71 ± 0.97	1.26 ± 0.57	1.65 ± 0.82	2.22 ± 1.17	<0.001
Uric acid, µmol/L	372.63 ± 96.58	365.08 ± 95.97	371.80 ± 92.73	380.94 ± 100.84	0.358
Albumin, mmol/L	39.75 ± 3.79	39.51 ± 3.40	40.15 ± 3.76	39.58 ± 4.15	0.273
FBG, mmol/L	6.39 ± 2.36	4.80 ± 0.75	5.80 ± 1.15	8.55 ± 2.69	<0.001
TyG index	8.91 ± 0.66	8.38 ± 0.42	8.83 ± 0.44	9.50 ± 0.55	<0.001
AIP	0.20 ± 0.28	0.02 ± 0.22	0.19 ± 0.24	0.37 ± 0.25	<0.001
METS-IR	39.65 ± 6.13	35.79 ± 5.55	40.15 ± 5.04	42.98 ± 5.51	<0.001
eGFR, mL/min/1.73 m^2^	94.74 (80.62, 111.23)	95.27 (79.74, 106.47)	94.21 (82.43, 107.28)	95.72 (79.55, 114.38)	0.857
D-dimer, mg/L	0.26 (0.16, 0.54)	0.26 (0.17,0.55)	0.24 (0.15,0.40)	0.33 (0.19,0.65)	0.029
CRP ≥ 5 mg/L	136 (29.96)	44 (29.14)	44 (29.14)	48 (31.58)	0.866
NT-proBNP ≥ 70 ng/L	405 (89.21)	133 (88.08)	132 (87.42)	140 (92.11)	0.363
Diagnosis, *n* (%)					<0.001
UA	163 (35.90)	72 (47.68)	61 (40.40)	30 (19.74)	
NSTEMI	148 (32.60)	55 (36.42)	41 (27.15)	52 (34.21)	
STEMI	143 (31.50)	24 (15.89)	49 (32.45)	70 (46.05)	
Angiography					
Gensini score	50.00 (32.00, 84.00)	42.00 (23.50,73.50)	50.00 (31.00,79.50)	66.00 (40.00,95.25)	<0.001
Multivessel disease, *n* (%)	325 (71.59)	97 (64.24)	103 (68.21)	125 (82.24)	0.001
Intervention					
LM	9 (1.98)	3 (1.99)	5 (3.31)	1 (0.66)	0.210
LAD	307 (67.62)	103 (68.21)	105 (69.54)	99 (65.13)	0.702
LCX	157 (34.58)	54 (35.76)	47 (31.13)	56 (36.84)	0.540
RCA	212 (46.70)	58 (38.41)	73 (48.34)	81 (53.29)	0.030
Multiple stents (*n* ≥ 2), *n* (%)	263 (57.93)	88 (58.28)	86 (56.95)	89 (58.55)	0.956
Mean stent diameter, mm	3.00 (2.75, 3.38)	3.00 (2.75, 3.25)	3.00 (2.75, 3.50)	3.00 (2.75, 3.25)	0.819
Length of stents, mm	48.00 (29.00, 72.00)	48.00 (28.00, 64.50)	46.00 (27.00, 70.00)	49.50 (30.00, 91.25)	0.137
Medications at discharge, *n* (%)					
P2Y12 inhibitor^a^					
Clopidogrel	129 (28.41)	50 (33.11)	47 (31.13)	32 (21.05)	0.044
Ticagrelor	325 (71.59)	101 (66.89)	104 (68.87)	120 (78.95)	0.044
Ezetimibe	113 (24.89)	34 (22.52)	40 (26.49)	39 (25.66)	0.701
Statin intensity^b^					
High-intensity statin	150 (33.04)	48 (31.79)	52 (34.44)	50 (32.89)	0.886
Non-high-intensity statin	310 (66.96)	103 (68.21)	99 (65.56)	102 (67.11)	0.886
Beta-blocker	344 (75.77)	107 (70.86)	113 (74.83)	124 (81.58)	0.089
ACEI/ARB	237 (52.20)	86 (56.95)	79 (52.32)	72 (47.37)	0.248
Spironolactone	64 (14.10)	17 (11.26)	17 (11.26)	30 (19.74)	0.050
Insulin	35 (7.71)	4 (2.65)	5 (3.31)	26 (17.11)	<0.001
Other hypoglycemic agents	127 (27.97)	23 (15.23)	27 (17.88)	77 (50.66)	<0.001
Calcium channel blockers	95 (20.97)	41 (27.15)	35 (23.18)	19 (12.58)	0.006
Diuretics	64 (14.10)	18 (11.92)	14 (9.27)	32 (21.05)	0.008
ISR	70 (15.12)	14 (9.27)	26 (17.22)	30 (19.14)	0.031

CHG, cholesterol, high-density lipoprotein,glucose index; BMI, body mass index; LVEF, left ventricular ejection fraction; LDL-C, low-density lipoprotein-cholesterol; TC, total cholesterol; HDL-C, high-density lipoprotein-cholesterol; FBG, fasting blood glucose; TyG, triglyceride-glucose index; AIP, atherogenic index of plasma; METS-IR, metabolic score for insulin resistance; eGFR, estimated glomerular filtration rate; CRP, C reactive protein; NT-proBNP, N-terminal pro-brain natriuretic peptide; UA, unstable angina; NSTEMI, non ST-segment elevation myocardial infarction; STEMI, ST-segment elevation myocardial infarction; LM, left main artery; LAD, left anterior descending artery; LCX, left circumflex artery; RCA, right coronary artery; ACEI/ARB, angiotensin-converting enzyme inhibitor/angiotensin receptor blocker; ISR, in-stent restenosis.

a All patients received dual antiplatelet therapy with aspirin in combination with either ticagrelor or clopidogrel.

b Statin intensity was categorized as high-intensity (atorvastatin 40–80 mg/day or rosuvastatin 20–40 mg/day) or non-high-intensity (all other regimens).

According to tertiles of the CHG index, all participants were categorized into three groups (Table 1), T1 (CHG index <5.264, *n* = 151), T2 (5.264 ≤ CHG index <5.666, *n* = 151), and T3 (CHG index ≥5.666, *n* = 152). With increasing tertiles of the CHG index, levels of serum FBG, TG, TC, LDL-C, TyG index, AIP, METS-IR, prevalence of hypercholesteraemia, STEMI, and diabetes, BMI, and use of insulin, hypoglycemic agents, and diuretics all showed significant increases (*P* < 0.05). Nevertheless, patients with higher CHG indices were relatively younger, had lower HDL-C levels, LVEF, and exhibited lower rates of calcium channel blocker (CCB) use. No significant differences were observed in other parameters among the three groups (*P* > 0.05). Notably, there was a trend toward higher rates of multi-vessel disease, target vessel in RCA, and Gensini score, as well as a significantly greater incidence of ISR, in the higher CHG index groups.

### CHG index and the prevalence of ISR after successful DES-based PCI

**Figure 1:**
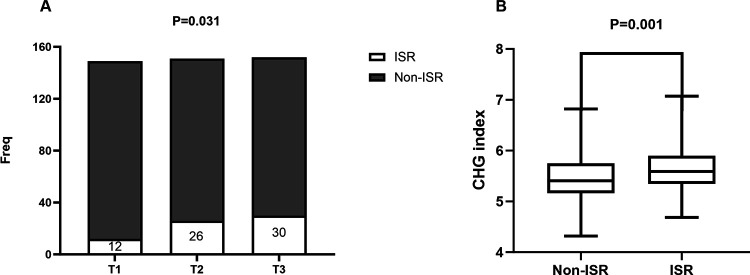
**(A)** The impacts of the CHG index on the prevalence of DES-ISR in the overall study.DES, drug-eluting stent; ISR, in-stent restenosis. **(B)** The comparison of the CHG index level between the ISR and non-ISR groups in the overall study population. CHG index, cholesterol,high-density lipoprotein,glucose index; ISR, in-stent restenosis.

As shown in Figure 1A, the prevalence of ISR had stepwise increase with the increasing tertile of the CHG index (9.27% vs. 17.22% vs. 19.14%; *P* = 0.031). Additionally, it is noteworthy that the ISR group also had a significantly higher CHG index than the non-ISR group (5.47 ± 0.46 vs. 5.66 ± 0.47, *P* = 0.001, Figure 1B).

### Association of the CHG index with the risk of DES-ISR in univariate analysis and multivariable analysis

In the univariate logistic regression analysis, the CHG index, as a continuous variable, was positively associated with the risk of ISR after successful DES-PCI (OR =  2.43, 95% CI 1.42–4.16, *P* = 0.001; Table 2). When analyzed categorically, the risk of ISR was significantly higher in the second (OR = 2.04; 95% CI, 1.02–4.07; *P* = 0.045) and third tertiles (OR = 2.41; 95% CI, 1.25–4.75; *P* = 0.011; Table 2) compared to the first tertile.

**Table 2:** Logisticregression analyses for the association between CHG index and DES-ISR.

	OR	95% CI	*P*-value
Unjusted			
CHG, per 1-unit increase	2.43	1.42 to 4.16	0.001
Tertile 1	Reference		
Tertile 2	2.04	1.02 to 4.07	0.045
Tertile 3	2.41	1.22 to 4.75	0.011
Model 1			
CHG, per 1-unit increase	2.45	1.42 to 4.23	0.001
Tertile 1	Reference		
Tertile 2	2.18	1.08 to 4.42	0.031
Tertile 3	2.50	1.25 to 4.99	0.009
Model 2			
CHG, per 1-unit increase	2.51	1.26 to 4.99	0.009
Tertile 1	Reference		
Tertile 2	2.23	1.09 to 4.58	0.028
Tertile 3	2.32	1.05 to 5.12	0.037
Model 3			
CHG, per 1-unit increase	2.61	1.28 to 5.33	0.008
Tertile 1	Reference		
Tertile 2	2.33	1.12 to 4.85	0.024
Tertile 3	2.40	1.05 to 5.49	0.038

Model 1, adjusted for age, sex, and BMI.

Model 2, adjust for age, sex, BMI, LVEF, CRP, hypertension, diabetes mellitus, smoking, eGFR.

Model 3, adjust for age, sex, BMI, LVEF, CRP, hypertension, diabetes mellitus, smoking, eGFR, total length of stents, meanl stent diameter, Gensini scrore, multiple stents and multivessel disease.

CHG, cholesterol, high-density lipoprotein, glucose index; DES, drug eluting stent; ISR, in-stent restenosis; OR, odds ratio; CI, confidence interval; BMI, body mass index; LVEF, left ventricular ejection fraction; CRP, C reactive protein; eGFR, estimated glomerular filtration rate.

In multivariate logistic regression models, with the CHG index initially included as a continuous variable, each 1-unit increase in CHG index remained independently associated with elevated ISR risk in model 1 (OR = 2.45; 95% CI, 1.42–4.23; *P* = 0.001; Table 2), model 2 (OR = 2.51; 95% CI, 1.26–4.99; *P* = 0.009), and the fully adjusted model 3 (OR = 2.61; 95% CI, 1.28–5.33; *P* = 0.008).

This association persisted when the CHG index was evaluated as a categorical variable, in model 3, after comprehensive adjustment, the adjusted odds ratios (95% CI) for the second and third tertiles were 2.33 (1.12–4.85; *P* = 0.024) and 2.40 (1.05–5.49; *P* = 0.038), respectively, compared with the reference group (Table 3).

**Table 3:** Association between CHG index and other cardiovascular risk factors.

Variables	Correlation coefficient (r)	*P*-value
Age	−0.181	<0.001
BMI	0.041	0.379
eGFR	−0.23	0.619
Uric acid	0.087	0.063
LDL-C	0.365	<0.001
TG	0.447	<0.001
LVEF	−0.179	<0.001
TyG index	0.766	<0.001
AIP	0.570	<0.001
METS-IR	0.530	<0.001
Total length of stents	0.133	0.004
Mean stent diameter	0.009	0.847
Gensini score	0.243	<0.001

CHG, cholesterol,high-density lipoprotein,glucose index; BMI, body mass index; eGFR, estimated glomerular filtration rate; LDL-C, low-density-lipoprotein-cholesterol; TG, triglyceride; LVEF, left ventricular ejection fraction; TyG, triglyceride-glucose index; AIP, atherogenic index of plasma; METS-IR, metabolic score for insulin resistance.

### Subgroup and sensitivitanalysis

**Figure 2:**
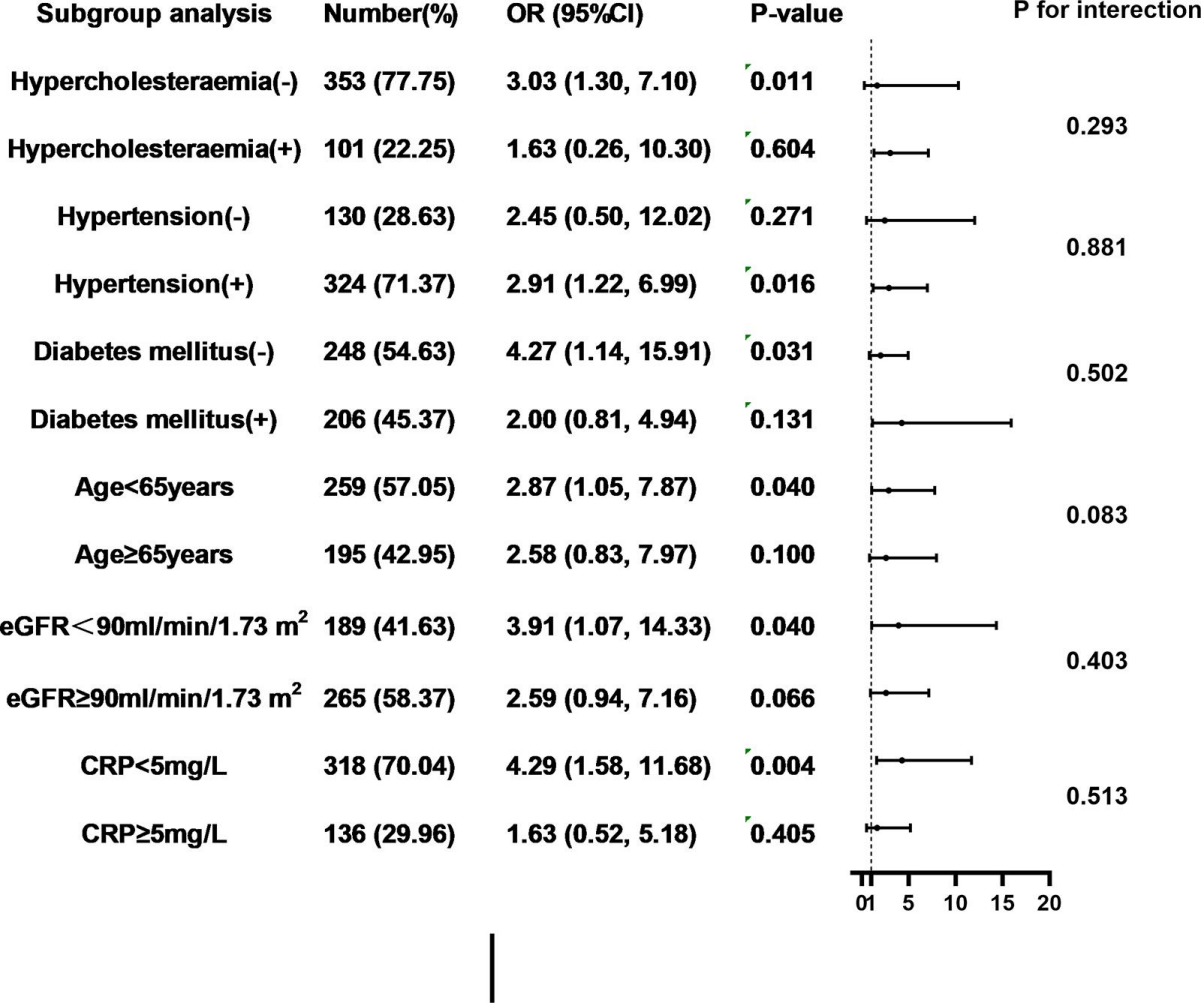
Forest plot investigating the association between the CHG index and the prevalence of DES-ISR in different subgroups. CHG index, cholesterol,high-density lipoprotein,glucose index; DES, drug-eluting stent; ISR, in-stent restenosis; CRP, C reactive protein; eGFR, estimated glomerular filtration rate; OR, odds ratio; CI, confidence interval.

The consistency of the association between the CHG index and DES-ISR was evaluated across key clinical subgroups (Figure 2). In multivariable-adjusted model 3, a positive association was observed in most subgroups. Notably, no significant interactions were identified between the CHG index and any subgroup variable (all *P* for interaction > 0.05). Sensitivity analyses confirmed the stability of the primary finding (Supplementary Table S4). The positive association between the CHG index and DES-ISR risk remained statistically significant across all models. In the model that additionally adjusted for statin intensity, ezetimibe use, and P2Y12 inhibitor type, each 1-unit increase in the CHG index continued to be independently associated with a higher risk of DES-ISR(OR = 2.61; 95% CI, 1.28–5.32; *P* = 0.008; Supplementary Table S4). And when the CHG index was evaluated as a categorical variable, the adjusted odds ratios (95% CI) for the second and third tertiles were 2.31 (1.11–4.84; *P* = 0.026) and 2.40 (1.05–5.49 == 51; *P* = 0.038 = 9), respectively, compared with the reference group (Supplementary Table S4).

### The predictive value of the CHG index for DES-ISR

**Figure 3:**
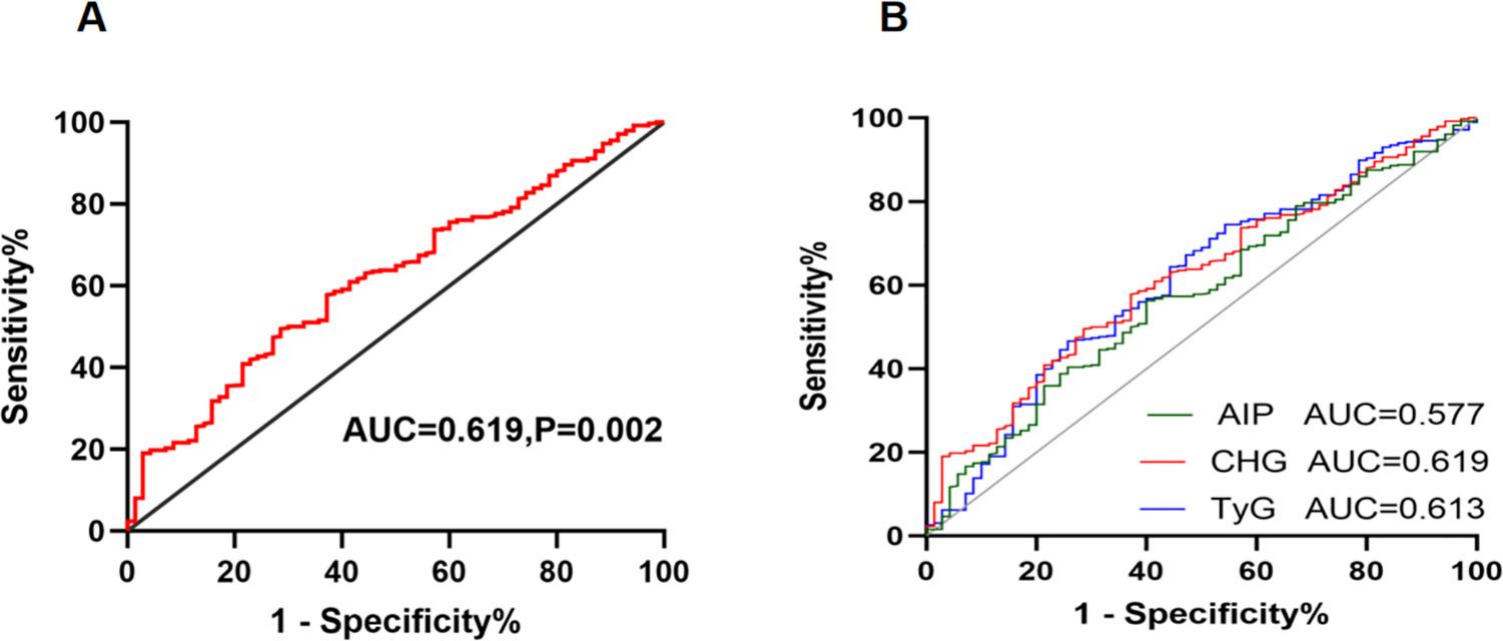
Receiver operating characteristic (ROC) curve analysis for predicting DES-ISR. **(A)** ROC curve of the CHG index alone. **(B)** Comparison of ROC curves between the CHG index and other metabolic indices (TyG index, AIP). CHG index, cholesterol,high-density lipoprotein,glucose index; DES, drug-eluting stent; ISR, in-stent restenosis; AUC, area under curve; AIP, atherogenic index of plasma; TyG index, triglyceride–glucose index.

**Figure 4:**
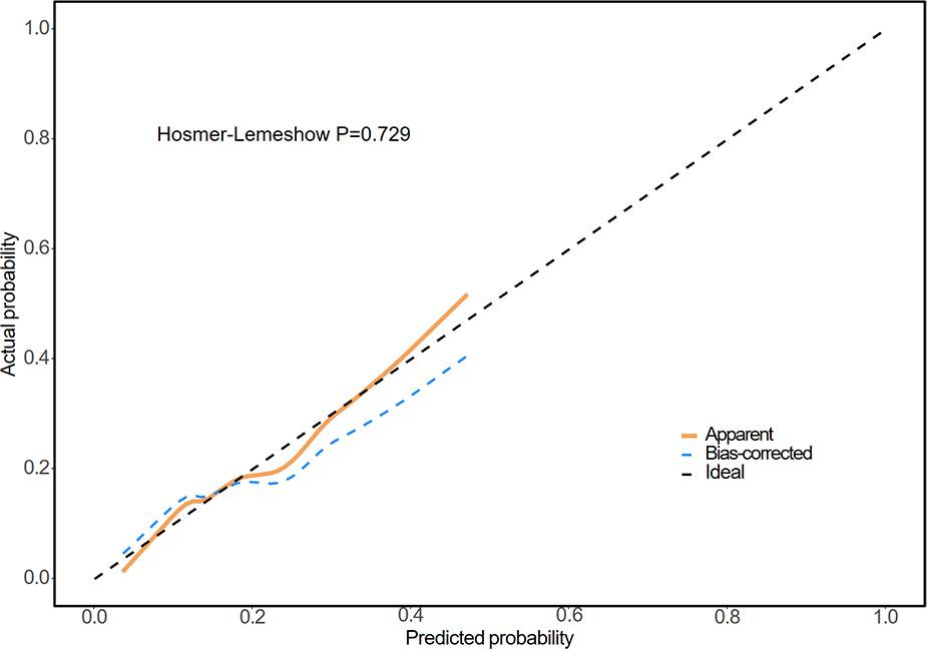
Calibration plot of the fully adjusted model (model 3) for predicting drug-eluting stent in-stent restenosis (DES-ISR). DES, drug-eluting stent; ISR, in-stent restenosis.

As presented in Figure 3A, the ROC curve analysis indicated that the CHG index provides modest predictive value for DES-ISR in patients with ACS, with an AUC of 0.619 (95% CI, 0.552 to 0.687, *P* = 0.002). The optimal cutoff value was 5.402 (sensitivity, 71.4%, specificity, 49.7%). The Hosmer-Lemeshow goodness-of-fit test for the fully adjusted model (Model 3) demonstrated excellent calibration (*P* = 0.729), indicating no significant deviation between the predicted probabilities of DES-ISR and the observed outcomes (Figure 4). The area under the receiver operating characteristic curve (AUC) for the CHG index predicting ISR was significantly higher compared to metabolic score for insulin resistance (METS-IR), (CHG index vs. METS-IR, 0.619 vs. 0.514, *P* = 0.009). Furthermore, the AUC for the CHG index was greater than that of TyG index (0.619 vs. 0.613) and AIP (0.619 vs. 0.577), but Delong test revealed this difference was not statistically significant (CHG index vs. TyG index, *P* = 0.811, CHG index vs. AIP, *P* = 0.236) (Figure 3B) (Additional File, Supplementary Tables S2, S3).

### Analysis of relationships between CHG and DES-ISR risk using RCS models

**Figure 5:**
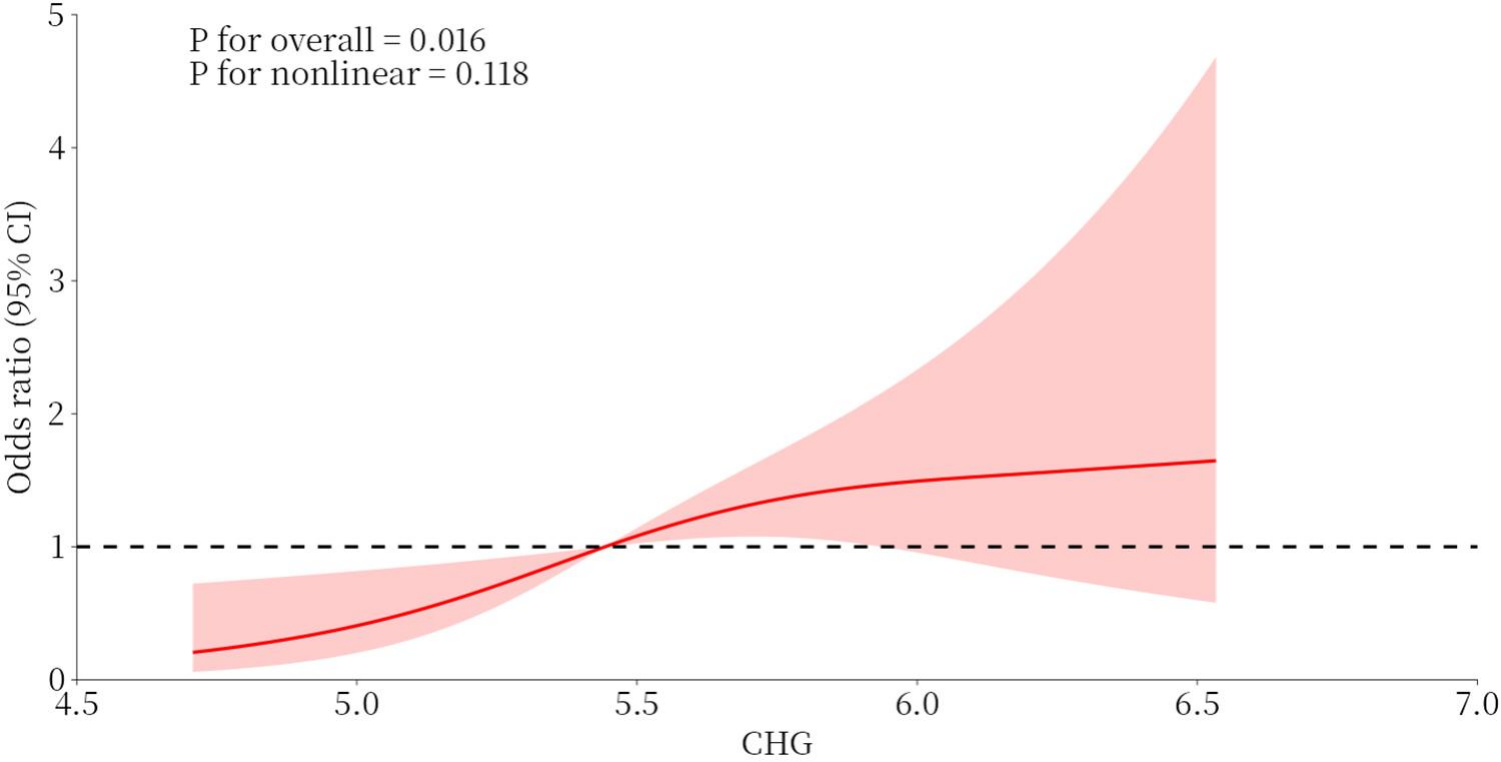
Restricted cubic spline models analyzed the relationship between CHG index and DES-ISR. CHG index, cholesterol,high-density lipoprotein,glucose index; DES, drug-eluting stent; ISR, in-stent restenosis.

Figure 5 illustrates that, in the unadjusted RCS model, the CHG index exhibited a positive linear association with the risk of ISR after DES-PCI in ACS patients (*P* = 0.006, *P* for nonlinear = 0.233). Even after adjusting for confounders, a significant linear relationship persisted between the CHG index and the risk of ISR (*P* for overall = 0.016; *P* for nonlinea*r* = 0.118).

### Association between CHG index and other cardiovascular risk factors

To further ascertain the association between CHG index and other risk factors for adverse cardiovascular outcomes, Spearman rank or Pearson's correlation analyses were performed (Table 3). The CHG index demonstrated a positive correlation with the Gensini scores (*r* = 0.243, *P* < 0.001), the TyG index (*r* = 0.766, *P* < 0.001), the AIP (*r* = 0.570, *P* < 0.001), the METS-IR (*r* = 0.530, *P* < 0.001), LDL-C (*r* = 0.365, *P* < 0.001), TG (*r* = 0.766, *P* < 0.001), and total stent lengths (*r* = 0.133, *P* = 0.004). In contrast, the CHG index demonstrated a negative correlation with age (*r* = −0.181, *P* < 0.001) and LVEF(*r* = −0.179, *P* < 0.001).

## Discussion

In this study, the CHG index demonstrated a significant, independent, and linear association with the risk of DES-ISR in patients with ACS. While its discriminatory capacity as a standalone marker was modest (AUC = 0.619), the fully adjusted model exhibited excellent calibration. Notably, its predictive performance was comparable to that of the established TyG index. This suggests that the CHG index, by integrating pathways of cholesterol metabolism and glucose homeostasis, provides complementary metabolic information of similar prognostic value for ISR risk stratification.

As a composite biomarker, the CHG index combines three conventional metabolic measures including TC, FBG, and HDL-C (24). This integration may better reflect systemic metabolic dysfunction and its impact on vascular pathology than individual components alone. Previous studies have confirmed that elevated fasting glucose, reduced HDL-C, and increased total cholesterol individually contribute to the progression of coronary artery disease (CAD) and to the risk of ISR after PCI in CADs patients (25–29). The findings extend the utility of the CHG index, previously associated with type 2 diabetes and cardiovascular events, to the prediction of ISR post-DES] (10, 30).

The mechanisms underlying the associations of the CHG index with ISR are not elucidated, it may be attributed to the synergistic effects of insulin resistance (IR) and low-grade inflammation on vascular remodeling post-stenting. First, a high CHG index may reflects underlying IR, which disrupts the balance of vascular insulin signaling. The protective PI3K/Akt pathway (stimulating endothelial nitric oxide production to inhibit smooth muscle cell growth) is attenuated, while the proliferative MAPK/ERK pathway is relatively overactive (31–33). This shift creates a microenvironment favoring vascular smooth muscle cell (VSMC) proliferation and migration—key events in neointimal hyperplasia.Second, the dysmetabolic state indicated by the CHG index (hyperglycemia, dyslipidemia) promotes a chronic pro-inflammatory and pro-thrombotic condition (34). This amplifies the vascular injury response after stenting, leading to sustained cytokine release and increased platelet reactivity. The resulting inflammation and micro-thrombosis on stent struts further stimulate VSMC proliferation and extracellular matrix deposition, directly accelerating neointima formation (32, 35–37). Furthermore, the CHG index may indicate a prothrombotic state, since hyperglycemia and dyslipidemia intensify platelet adhesion and aggregation while elevating fibrinogen levels. The hypercoagulable state contributes to in-stent restenosis by triggering a cascade of pathological events, including accelerated thrombus formation, amplified inflammatory responses, enhanced vascular smooth muscle cell proliferation and migration, and impaired endothelial repair, which collectively drive neointimal hyperplasia and luminal narrowing (38, 39). In summary, the CHG index captures a metabolic profile that promotes ISR through concurrent impairment of protective insulin signaling and amplification of inflammatory-thrombotic pathways, collectively driving pathological vascular remodeling.

Notably, the association persisted despite patients receiving guideline-directed therapy, including high-intensity statins. This suggests the CHG index may identify residual cardiometabolic risk not fully addressed by conventional LDL-C lowering. It particularly highlights persistent abnormalities in glucose metabolism and HDL functionality, pointing to potential therapeutic targets beyond standard lipid management.

Clinically, the CHG index is easily calculated from routine laboratory tests, offering a low-cost tool for risk assessment. The study revealed a linear, dose-response relationship between the CHG index and DES-ISR risk. The cut-off of 5.402, derived from the maximum Youden's index (sensitivity 71.4%, specificity 49.7%), may serve as a practical threshold for risk stratification. Its higher sensitivity relative to specificity implies that the index was more useful for identifying patients unlikely to develop ISR than for confirming those at high risk. In practice, a CHG index below 5.402 could support a decision for routine post-PCI surveillance. On the other hand, a value ≥5.402 should alert clinicians to review and intensify management of underlying metabolic disturbances—particularly glucose control and HDL-C levels—and to consider closer clinical follow-up. Whether such a strategy ultimately improves outcomes must be tested in prospective, intervention-driven studies.

The current research has certain limitations that should be acknowledged. First, its retrospective, single-center design with a modest sample size limits causal inference and generalizability. Second, the predominantly male cohort (81.5%) may restrict the applicability of findings to female patients. Third, follow-up angiography was performed within a heterogeneous time window (6–24 months); although follow-up duration was not associated with ISR occurrence (*P* = 0.136), time-stratified analyses were not conducted. Fourth, the CHG index was measured only at baseline, precluding assessment of the impact of metabolic changes over time. Fifth, detailed data on lesion characteristics (e.g., calcification, bifurcation) and stent generation/model were not collected; these factors could confound the observed association. Sixth, all patients received guideline-directed pharmacotherapy (including statins), meaning the findings reflect residual risk within a treated population. Future multicenter, prospective studies with protocol-defined angiographic timepoints, intracoronary imaging, serial metabolic assessments, and balanced sex representation are needed to validate and extend these results.

## Conclusion

An elevated CHG index—comprising total cholesterol, glucose, and high-density lipoprotein cholesterol—is independently associated with the risk of in-stent restenosis in patients after DES-PCI with ACS.

## Data Availability

The raw data supporting the conclusions of this article will be made available by the authors, without undue reservation.
