# Supplementary material for: Cholesterol, high-density lipoprotein, glucose index as a novel marker for predicting in-stent restenosis after drug-eluting stent implantation in patients with acute coronary syndrome
Source: Front Cardiovasc Med. 2026 Feb 3;13:1740216. doi: 10.3389/fcvm.2026.1740216 (PMC12909208; doi:10.3389/fcvm.2026.1740216)
Supplement: Supplementary file 1 [file Datasheet1.doc]

**Table S1 Univariate logisticregression analyses for the association between CHG index and DES-ISR**

| Variables | β | S.E | Z | *P* | OR (95%CI) |
| --- | --- | --- | --- | --- | --- |
|
| Diagnosis |  |  |  |  |  |
| UA |  |  |  |  | 1.00 (Reference) |
| NSTEMI | -0.20 | 0.30 | -0.67 | 0.503 | 0.82 (0.45 ~ 1.48) |
| STEMI | -0.51 | 0.33 | -1.57 | 0.117 | 0.60 (0.31 ~ 1.14) |
| Gender |  |  |  |  |  |
| Female |  |  |  |  | 1.00 (Reference) |
| Male | -0.22 | 0.32 | -0.68 | 0.494 | 0.80 (0.43 ~ 1.50) |
| Hypertension |  |  |  |  |  |
| No |  |  |  |  | 1.00 (Reference) |
| Yes | 0.35 | 0.31 | 1.16 | 0.247 | 1.42 (0.78 ~ 2.59) |
| Hypercholesteraemia |  |  |  |  |  |
| No |  |  |  |  | 1.00 (Reference) |
| Yes | 0.14 | 0.30 | 0.45 | 0.656 | 1.15 (0.63 ~ 2.08) |
| Previous stroke |  |  |  |  |  |
| No |  |  |  |  | 1.00 (Reference) |
| Yes | 0.19 | 0.51 | 0.37 | 0.713 | 1.21 (0.44 ~ 3.29) |
| Diabetes mellitus |  |  |  |  |  |
| No |  |  |  |  | 1.00 (Reference) |
| Yes | 0.36 | 0.26 | 1.36 | 0.173 | 1.43 (0.86 ~ 2.38) |
| Smoking |  |  |  |  |  |
| No |  |  |  |  | 1.00 (Reference) |
| Yes | -0.48 | 0.26 | -1.84 | 0.066 | 0.62 (0.37 ~ 1.03) |
| Drinking |  |  |  |  |  |
| No |  |  |  |  | 1.00 (Reference) |
| Yes | -0.48 | 0.29 | -1.65 | 0.100 | 0.62 (0.35 ~ 1.10) |
| hyperuricemia |  |  |  |  |  |
| No |  |  |  |  | 1.00 (Reference) |
| Yes | -0.68 | 0.75 | -0.90 | 0.368 | 0.51 (0.12 ~ 2.22) |
| CRP≥5mg/L |  |  |  |  |  |
| No |  |  |  |  | 1.00 (Reference) |
| Yes | 0.60 | 0.27 | 2.26 | 0.024 | 1.83 (1.08 ~ 3.10) |
| NT-proBNP≥70ng/L |  |  |  |  |  |
| No |  |  |  |  | 1.00 (Reference) |
| Yes | 1.11 | 0.61 | 1.82 | 0.064 | 3.04 (0.92 ~ 10.06) |
| Intervention |  |  |  |  |  |
| LM |  |  |  |  |  |
| No |  |  |  |  | 1.00 (Reference) |
| Yes | 0.46 | 0.81 | 0.57 | 0.571 | 1.58 (0.32 ~ 7.79) |
| LCX |  |  |  |  |  |
| No |  |  |  |  | 1.00 (Reference) |
| Yes | -0.02 | 0.27 | -0.06 | 0.955 | 0.98 (0.58 ~ 1.68) |
| LAD |  |  |  |  |  |
| No |  |  |  |  | 1.00 (Reference) |
| Yes | 0.38 | 0.29 | 1.29 | 0.197 | 1.46 (0.82 ~ 2.60) |
| RCA |  |  |  |  |  |
| No |  |  |  |  | 1.00 (Reference) |
| Yes | -0.11 | 0.26 | -0.44 | 0.660 | 0.89 (0.53 ~ 1.49) |
| Multiple stents |  |  |  |  |  |
| No |  |  |  |  | 1.00 (Reference) |
| Yes | 0.17 | 0.27 | 0.64 | 0.519 | 1.19 (0.70 ~ 2.00) |
| Medications at discharge |  |  |  |  |  |
| P2Y12 inhibitor^a^ |  |  |  |  |  |
| clopidogrel |  |  |  |  |  |
| No |  |  |  |  | 1.00 (Reference) |
| Yes | -0.44 | 0.31 | -1.40 | 0.161 | 0.65 (0.35 ~ 1.19) |
| ticagrelor |  |  |  |  |  |
| No |  |  |  |  |  |
| Yes | 0.44 | 0.31 | 1.40 | 0.61 | 1.55 (0.84 ~ 2.85) |
| ezetimibe |  |  |  |  |  |
| No |  |  |  |  | 1.00 (Reference) |
| Yes | 0.22 | 0.29 | 0.77 | 0.439 | 1.25 (0.71 ~ 2.21) |
| high-intensity statin^a^ |  |  |  |  |  |
| No |  |  |  |  | 1.00 (Reference) |
| Yes | 0.07 | 0.27 | 0.24 | 0.810 | 1.07 (0.62 ~ 1.83) |
| ACEI/ARB |  |  |  |  |  |
| No |  |  |  |  | 1.00 (Reference) |
| Yes | 0.24 | 0.26 | 0.90 | 0.369 | 1.27 (0.76 ~ 2.12) |
| Beta-block |  |  |  |  |  |
| No |  |  |  |  | 1.00 (Reference) |
| Yes | 0.39 | 0.33 | 1.20 | 0.232 | 1.48 (0.78 ~ 2.82) |
| Insulin |  |  |  |  |  |
| No |  |  |  |  | 1.00 (Reference) |
| Yes | 0.53 | 0.43 | 1.26 | 0.209 | 1.71 (0.74 ~ 3.93) |
| Spironolactone |  |  |  |  |  |
| No |  |  |  |  | 1.00 (Reference) |
| Yes | -0.12 | 0.39 | -0.32 | 0.746 | 0.88 (0.41 ~ 1.88) |
| Other hypoglycemic agents |  |  |  |  |  |
| No |  |  |  |  | 1.00 (Reference) |
| Yes | 0.50 | 0.27 | 1.85 | 0.065 | 1.66 (0.97 ~ 2.83) |
| Diuretics |  |  |  |  |  |
| No |  |  |  |  | 1.00 (Reference) |
| Yes | 0.15 | 0.36 | 0.42 | 0.673 | 1.16 (0.57 ~ 2.36) |
| Calcium channel blockers |  |  |  |  |  |
| No |  |  |  |  | 1.00 (Reference) |
| Yes | 0.50 | 0.29 | 1.69 | 0.092 | 1.64 (0.92 ~ 2.92) |
| Multivessel disease |  |  |  |  |  |
| No |  |  |  |  | 1.00 (Reference) |
| Yes | -0.09 | 0.28 | -0.32 | 0.749 | 0.91 (0.52 ~ 1.59) |
| Age | -0.00 | 0.01 | -0.08 | 0.934 | 1.00 (0.97 ~ 1.02) |
| BMI | -0.05 | 0.05 | -1.01 | 0.312 | 0.95 (0.87 ~ 1.05) |
| LDL | 0.15 | 0.12 | 1.28 | 0.200 | 1.16 (0.92 ~ 1.45) |
| TC | 0.17 | 0.11 | 1.50 | 0.135 | 1.18 (0.95 ~ 1.47) |
| Albumin | -0.01 | 0.03 | -0.36 | 0.717 | 0.99 (0.92 ~ 1.06) |
| Uric acid | -0.00 | 0.00 | -1.07 | 0.283 | 1.00 (1.00 ~ 1.00) |
| HDL | -0.61 | 0.53 | -1.17 | 0.242 | 0.54 (0.19 ~ 1.51) |
| ALT | 0.00 | 0.00 | 0.02 | 0.983 | 1.00 (0.99 ~ 1.01) |
| AST | -0.00 | 0.00 | -0.89 | 0.373 | 1.00 (1.00 ~ 1.00) |
| eGFR | -0.00 | 0.00 | -0.76 | 0.447 | 1.00 (0.99 ~ 1.01) |
| FBG | 0.11 | 0.05 | 2.21 | **0.027** | 1.11 (1.01 ~ 1.23) |
| TyG index | 0.54 | 0.19 | 2.81 | **0.005** | 1.72 (1.18 ~ 2.52) |
| D-dimer | 0.10 | 0.06 | 1.70 | 0.089 | 1.10 (0.99 ~ 1.23) |
| LVEF | -0.00 | 0.01 | -0.33 | 0.745 | 1.00 (0.97 ~ 1.02) |
| Gensini score | 0.00 | 0.00 | 0.67 | 0.500 | 1.00 (1.00 ~ 1.01) |
| Length of stents | 0.01 | 0.00 | 2.22 | **0.026** | 1.01 (1.01 ~ 1.01) |
| Mean stent diameter | -0.40 | 0.33 | -1.21 | 0.226 | 0.67 (0.35 ~ 1.28) |
| Interval from index PCI to follow-up angiography, months | 0.04 | 0.03 | 1.49 | 0.136 | 1.04 (0.99~ 1.99) |

^a^All patients received dual antiplatelet therapy with aspirin in combination with either ticagrelor or clopidogrel;^b^Statin intensity was categorized as high-intensity (atorvastatin 40-80 mg/day or rosuvastatin 20-40 mg/day) or non-high-intensity (all other regimens)

CHG, cholesterol,high-density lipoprotein,glucose index; DES, drug eluting stent; ISR, in-stent restenosis; OR, odds ratio; CI, confidence interval; UA, unstable angina; NSTEMI, non ST-segment elevation myocardial infarction; STEMI, ST-segment elevation myocardial infarction; CRP, C reactive protein; NT-proBNP, N-terminal pro-brain natriuretic peptide; LM, left main artery; LAD, left anterior descending artery; LCX, left circumflex artery; RCA, right coronary artery; ACEI/ARB, angiotensin-converting enzyme inhibitor/angiotensin receptor blocker; BMI, body mass index; LDL-C, low-density lipoprotein-cholesterol; TC, total cholestero; HDL-C, high-density lipoprotein-cholesterol; eGFR, estimated glomerular filtration rate; FBG, fasting blood glucose; TyG, triglyceride-glucose index; LVEF, left ventricular ejection fraction; PCI, Percutaneous Coronary Intervention

***Table S2 Comparisons of the area under the ROC curves of the CHG index, AIP, METS-IR and TyG index***

| variable | δAUC | 95% CI | z | p value |
| --- | --- | --- | --- | --- |
| CHG index vs. AIP | 0.042 | -0.028 to 0.113 | 1.186 | 0.236 |
| CHG index vs. METS-IR | 0.105 | 0.027 to 0.184 | 2.624 | 0.009 |
| CHG index vs. TyG index | 0.006 | -0.049 to 0.063 | 0.239 | 0.811 |

ROC curve, receiver operating characteristic curve; CHG, cholesterol,high-density lipoprotein,glucose index; AIP, atherogenic index of plasma; METS-IR, metabolic score for insulin resistence; TyG index, triglyceride–glucose index; AUC, aera under the curve; CI, confidence interval

***Table S3 ROC curve analysis of the CHG index, AIP, METS-IR and TyG index for in-stent restenosis***

| variable | AUC | 95% CI | sensitivity | specificity | P value | cut-off |
| --- | --- | --- | --- | --- | --- | --- |
| CHG index | 0.619 | 0.552 to 0.687 | 0.714 | 0.497 | 0.002 | 5.402 |
| AIP | 0.577 | 0.506 to 0.648 | 0.600 | 0.562 | 0.041 | 0.217 |
| TyG index | 0.613 | 0.541 to 0.684 | 0.743 | 0.466 | 0.003 | 8.759 |
| METS-IR | 0.514 | 0.438 to 0.590 | / | / | 0.704 | / |

ROC, curve, receiver operating characteristic curve; CHG, cholesterol,high-density lipoprotein,glucose index; AIP, atherogenic index of plasma; METS-IR, metabolic score for insulin resistence; TyG index, triglyceride–glucose index; AUC, aera under the curve; CI, confidence interval

**Table S4 Sensitivity analysis of the association between the CHG index and DES-ISR**

|  | **OR** | **95%CI** | **p-value** |
| --- | --- | --- | --- |
| Full model ＋ statin intensity^a^ |  |  |  |
| CHG,per 1-unit increase | 2.60 | 1.28 to 5.31 | 0.009 |
| Tertile 1 | Reference |  |  |
| Tertile 2 | 2.32 | 1.11 to 4.84 | 0.025 |
| Tertile 3 | 2.40 | 1.05 to 5.48 | 0.038 |
| Full model + ezetimibe use |  |  |  |
| CHG, per 1-unit increase | 2.65 | 1.30 to 5.39 | 0.007 |
| Tertile 1 | Reference |  |  |
| Tertile 2 | 2.33 | 1.12 to 4.88 | 0.024 |
| Tertile 3 | 2.44 | 1.07 to 5.58 | 0.034 |
| Full model +P2Y12 inhibitor type^b^ |  |  |  |
| CHG, per 1-unit increase | 2.60 | 1.27 to 5.31 | 0.009 |
| Tertile 1 | Reference |  |  |
| Tertile 2 | 2.33 | 1.12 to 4.86 | 0.024 |
| Tertile 3 | 2.39 | 1.04 to 5.49 | 0.040 |
| Full mode + statin intensity and P2Y12 inhibitor type |  |  |  |
| CHG, per 1-unit increase | 2.58 | 1.26 to 5.28 | 0.009 |
| Tertile 1 | Reference |  |  |
| Tertile 2 | 2.32 | 1.11 to 4.84 | 0.026 |
| Tertile 3 | 2.38 | 1.03 to 5.46 | 0.041 |
| Full mode + statin intensity, ezetimibe use and P2Y12 inhibitor type |  |  |  |
| CHG, per 1-unit increase | 2.61 | 1.28 to 5.32 | 0.008 |
| Tertile 1 | Reference |  |  |
| Tertile 2 | 2.31 | 1.11 to 4.84 | 0.026 |
| Tertile 3 | 2.40 | 1.05 to 5.51 | 0.039 |

Full model, adjust for age, sex, BMI, LVEF, CRP, hypertension, diabetes mellitus,smoking, eGFR, total length of stents, meanl stent diameter, Gensini scrore, multiple stents and multivessel disease

^a^Statin intensity was categorized as high-intensity (atorvastatin 40-80 mg/day or rosuvastatin 20-40 mg/day) or non-high-intensity (all other regimens); ^b^All patients received dual antiplatelet therapy with aspirin in combination with either ticagrelor or clopidogrel

CHG, cholesterol,high-density lipoprotein,glucose index; DES, drug eluting stent; ISR, in-stent restenosis; OR, odds ratio; CI, confidence interval; BMI, body mass index; LVEF, left ventricular ejection fraction; CRP, C reactive protein; eGFR, estimated glomerular filtration rate
